# Subject-Reported Satisfaction After Cell-Enriched Lipotransfer (CELT) for Lip Augmentation

**DOI:** 10.1007/s00266-025-04875-z

**Published:** 2025-05-19

**Authors:** Sophia Diesch, Konstantin Frank, Vanessa Brébant, Sebastian Bohusch, Eva Brix, Rui Zeng, Lukas Prantl

**Affiliations:** 1 Department of Plastic, Hand and Reconstructive Surgery, University Hospital Regensburg, Regensburg, Germany; 2 Department for Hand, Plastic and Aesthetic Surgery, Ludwig – Maximilian University Munich, Munich, Germany

**Keywords:** CELT, Lipotransfer, Lip augmentation, Lips, Autologous fat grating, Stem cells, Mesenchymal stem cells

## Abstract

**Background:**

Lip augmentation is a highly demanded cosmetic procedure due to the association of full lips with youth, beauty, and sensuality. Among various techniques, autologous fat grafting, particularly with the cell-enriched lipotransfer (CELT) method, has emerged as a natural and long-lasting option. Unlike hyaluronic acid fillers, which require regular touch-ups, CELT offers sustained volume retention and potentially higher patient satisfaction.

**Objective:**

This study aimed to assess patient satisfaction following lip augmentation using the CELT technique and compare it with traditional fat preparation methods, such as sedimentation and filtration. The study also sought to evaluate the influence of other variables, including gender, age, and fat injection volume, on satisfaction outcomes.

**Materials and Methods:**

A retrospective chart review of patients who underwent lip augmentation with autologous fat grafting was conducted. Satisfaction was measured using the FACE-Q^TM^ questionnaire, focusing on various lip-related aspects such as shape, fullness, and how well the lips suited the face. The study compared satisfaction outcomes between the CELT method and combined sedimentation/filtration techniques. Statistical analysis using the Kruskal–Wallis test and Pearson's correlation was performed to assess the influence of preparation method, injected volume, and patient demographics.

**Results:**

The CELT method consistently produced higher satisfaction compared to sedimentation and filtration (*p* < 0.001 for all items). Patients treated with CELT reported greater improvements in the shape of the lips, fullness of the lower lip, and Cupid’s bow. Gender and age were also significant factors, with males showing greater satisfaction in smile-related aspects, while females were more satisfied with the lip style. Older patients reported greater improvements in how well their lips suited their face, while higher injected volumes positively correlated with lip fullness and appearance.

**Conclusion:**

The CELT fat grafting technique significantly enhances patient satisfaction in lip augmentation compared to traditional fat preparation methods. CELT's higher fat retention and more natural results make it a superior option for lip augmentation. However, further studies incorporating objective volumetric measurements are needed to validate these findings.

**Level of Evidence IV:**

This journal requires that authors assign a level of evidence to each article. For a full description of these Evidence-Based Medicine ratings, please refer to the Table of Contents or the online Instructions to Authors www.springer.com/00266.

## Introduction

Lip augmentation has become a highly sought-after cosmetic procedure, driven by a combination of aesthetic desires and societal perceptions [1]. Full lips have long been associated with beauty, youth, and health, making them a symbol of vitality, fertility and attractiveness. As individuals age, the lips lose volume and definition, leading to a more aged appearance [2–4]. This natural process, coupled with the societal emphasis on youthful and full lips, has resulted in a growing demand for lip augmentation procedures aimed at rejuvenation and beautification.

There are several methods available for lip enhancement, each varying in invasiveness and longevity. At the most basic level, cosmetic products like lipstick and lip liner allow individuals to create the illusion of fuller lips. However, for more substantial and longer-lasting results, soft tissue fillers, such as hyaluronic acid (HA)-based fillers, are commonly used. These fillers temporarily increase lip volume and offer flexibility, as they are partially reversible and require minimal downtime. However, frequent touch-ups are needed to maintain the desired effect, as the fillers are gradually absorbed by the body [5, 6].

Among the more permanent solutions, autologous fat grafting, or lipofilling, stands out as a natural and long-lasting option. In this procedure, fat from the patient’s own body is used to augment the lips, making it a more organic solution, as “like replaces like”. Fat grafting offers the advantage of not introducing foreign materials into the body, thus minimizing the risk of allergic reactions or complications associated with synthetic fillers [7]. However, one significant challenge with fat grafting is the dynamic nature of the perioral region. The lips are in constant motion due to activities like speaking, eating, and facial expressions, which imposes mechanical stress on the adipocytes (fat cells) and can impact graft survival rates [8, 9].

Despite these challenges, advancements in fat harvesting and preparation techniques have improved the outcomes of fat grafting for lip augmentation. The cell-enriched lipotransfer (CELT) technique, for example, offers a more refined approach to fat grafting by enhancing the survival and integration of the grafted fat. CELT and CELT^PLUS^ (Cleaned and Enriched Lipid Tissue, Purified Long-lasting Ultra-concentrated Supergraft) are advanced techniques for the preparation of lipoaspirate tissue, each optimized for specific clinical indications. CELT involves a single centrifugation at 1600 RCF for 2 minutes, followed by the removal of lipid and aqueous phases, resulting in purified adipose tissue. This process is primarily used for volume grafting, such as in breast, gluteal augmentation and specific regions in the face, where the goal is to restore or enhance volume in a stable and biocompatible manner. The focus of CELT is on producing a tissue graft that maintains structural integrity while promoting long-term volume retention.

On the other hand, CELT^PLUS^ represents a more intensive process aimed at regenerative grafting. After initial inter-syringe processing, the tissue undergoes a second centrifugation at the same speed (1600 RCF for 2 minutes), during which a clear separation into three distinct phases (aqueous, lipid, and solid tissue) becomes visible. The removal of lipid and aqueous phases results in a highly concentrated graft, often referred to as a supergraft. This ultra-purified form is rich in viable cells, including stromal vascular fractions and regenerative cells, making it particularly suitable for regenerative purposes such as tissue repair and healing. CELT^PLUS^ supergrafts are frequently applied in procedures aiming to regenerate damaged tissues, promote wound healing, or improve aesthetic outcomes through tissue rejuvenation [10, 11]. This method has shown promising results, with higher take rates and more predictable long-term outcomes compared to traditional fat grafting methods.

Patient satisfaction is an essential aspect of any cosmetic procedure, and lip augmentation is no exception. The subjective perception of the results is often the most important determinant of success. This study aims to retrospectively assess the patient satisfaction following lip augmentation using the CELT technique and compare it to other fat preparation techniques.

## Materials and Methods

### Chart Review

A retrospective chart review was conducted to collect relevant patient data from individuals who underwent lip augmentation using autologous fat grafting. Data were collected on each patient's gender, age, and body mass index (BMI). Information on the location of fat harvesting was recorded, and if multiple harvesting sites were used, the site with the largest aspirated volume was noted. Additionally, the volume of fat injected into the lips was documented along with the method of fat preparation (CELT^PLUS^, sedimentation or filtration). Any complications that occurred during or after the procedure were also recorded. These complications included infection, fat resorption, asymmetry, and other adverse effects associated with the procedure. Approval for the study was obtained from the Institutional Review Board (IRB) of the REDACTED, ensuring that the research adhered to ethical guidelines and patient confidentiality.

### Preparation of Fat

Three different types of fat preparation methods were analyzed in this study: sedimentation or filtration and CELT^PLUS^. For all methods, fat was harvested using a machine-assisted approach with the body-jet® evo system operating in lipocollection mode. This system maintains a continuous vacuum at − 500 mbar with an adjustable particle size, utilizing either a 3.8 mm (0.85 mm cluster size) or a 3.5 mm (0.65 mm cluster size) harvesting cannula. This closed system minimizes collateral damage to surrounding connective and subcutaneous fatty tissues, nerves, blood vessels, and lymphatic vessels. Additionally, the system ensures that the cannula does not clog during suction, even at low vacuum levels.

Fat sedimentation involved allowing the harvested fat to settle naturally, with residual fluids separating from the fat cells through gravity. This method reduced the fluid content and concentrated the fat for further processing. Fat filtration, in contrast, used a mechanical filtration system with a fine-pore filter membrane that separated intact fat cells from non-cellular components, including oils, tumescent solution, and cellular debris. The filtered fat was passed through multiple filter layers to ensure uniform particle size and optimal purity while preserving the integrity of the viable adipocytes.

The CELT^PLUS^ method builds on the basic CELT protocol. In the CELT process, fat is first subjected to sedimentation in a Lipocollector, which reduces the residual fluid content to 15–30%. The harvested fat is then centrifuged at 1600 rcf for 2 minutes using a swing-out rotor centrifuge (Hettich Rotofix 32 A). CELT^PLUS^ includes additional inter-syringe processing steps and a secondary centrifugation step to enhance the purity and concentration of the fat graft.

Regardless of the preparation method, the fat was injected into the target tissue using a homogeneous tunneling technique to ensure even distribution across all tissue layers. Care was taken to avoid the confluence of tunnels, with a transplant-to-recipient volume ratio not exceeding 1:4 to optimize graft retention. For CELT^PLUS^-prepared fat, a 1 ml Luer-Lock syringe equipped with a 20 G (1 mm OD) cannula was used, ensuring even distribution for optimal tissue integration and healing. The injection and motion rate for CELT^PLUS^ was maintained at 1 ml per > 4 cm to ensure precise placement and minimal tissue disruption.

### Patient Satisfaction Survey

Patients were surveyed using the “Satisfaction with Lips” questionnaire from the FACE-Q^TM^ suite, a validated patient-reported outcome measure. This questionnaire assesses various aspects of the patient's satisfaction with their lips, including size, shape, fullness, and symmetry, as well as how well the lips suit the overall face and how they appear during smiling and when relaxed. Responses are recorded on a Likert scale, ranging from "Very Dissatisfied" to "Very Satisfied." The FACE-Q is widely used in cosmetic and reconstructive surgery to evaluate patient satisfaction and perceived outcomes, providing comprehensive insights into subjective perceptions following lip augmentation procedures.

### Statistical Analysis

Statistical analysis was conducted using SPSS (IBM, Armonk, New York, USA). Descriptive statistics, such as means and standard deviations (SD), were calculated for continuous variables like age and BMI. Categorical variables, such as gender and the occurrence of complications, were presented as frequencies and percentages. A statistical analysis was performed to investigate the influence of various factors, including harvesting site, gender, age, and total injected volume, on postoperative patient satisfaction with lip augmentation. Satisfaction was measured using ordinal scores ranging from 1 (very dissatisfied) to 4 (very satisfied) for each satisfaction item. The difference between preoperative and postoperative satisfaction was calculated for each patient. The Kruskal–Wallis test was used to assess the influence of categorical variables such as harvesting location, smoking status, and gender on satisfaction changes. Correlation analysis (Pearson's correlation) was used to evaluate the relationship between continuous variables (age and total injected volume) and satisfaction changes. Significant findings were reported for *p* values less than 0.05.

## Results

A total of 98 participants were included in the study, consisting of 88 women and 10 men. The mean age of the participants was 60.0 years (SD = 11.65), ranging from 22 to 82 years. The mean BMI of the participants was 24.24 (SD = 4.01), with a range of 17.5–41.8. This demographic distribution highlights the diversity of the study population in terms of age, gender, and body composition, providing a comprehensive basis for evaluating the outcomes of the procedures. For the shape of the lower lip, satisfaction increased postoperatively, with no patients reporting dissatisfaction and 35 reporting being very satisfied (*p* < 0.001). Similar results were observed for how well the lips suited the face, where dissatisfaction decreased, and 31 patients were very satisfied (*p* < 0.001). Improvements were also noted in how nice the lips looked when smiling, with 37 patients reporting very satisfied (*p* < 0.001). Satisfaction with the fullness of the lower lip increased as well, with 39 patients being very satisfied postoperatively (*p* < 0.001). Other features, such as the style of the lips, Cupid’s bow, and fullness of the upper lip, also showed significant improvements in postoperative satisfaction, highlighting the effectiveness of the procedure. The complete results of the survey are given in Table 1.

**Table 1:** Pre- and postoperative satisfaction of subjects

Lip aspect	Preoperative very dissatisfied	Postoperative very dissatisfied	Preoperative somewhat dissatisfied	Postoperative somewhat dissatisfied	Preoperative somewhat satisfied	Postoperative somewhat satisfied	Preoperative very satisfied	Postoperative very satisfied	Wilcoxon test statistic	*P* value
The shape of your lower lip?	0	0	57	13	40	50	1	35	129.0	< 0.001
How well your lips suit your face?	20	0	43	19	35	48	0	31	80.0	< 0.001
How nice your lips look when you smile?	3	0	52	22	43	39	0	37	225.0	< 0.001
How full your lower lip looks?	11	0	60	19	27	40	0	39	132.0	< 0.001
The style of your lips (e.g., pouty, natural)	22	0	51	19	23	42	2	37	153.5	< 0.001
The shape of your upper lip?	11	0	58	15	29	45	0	38	168.0	< 0.001
How turned up your upper lip (Cupid’s bow) looks?	11	0	54	17	33	41	0	40	163.0	< 0.001
The size of your lips?	8	0	59	19	30	49	0	29	180.0	< 0.001
How the outer corners of your lips look when your face is relaxed (still)?	13	0	66	17	19	45	0	36	67.5	< 0.001
How full your upper lip looks?	5	0	52	16	41	55	0	27	150.0	< 0.001

### Influence of Harvesting Location on Satisfaction

Fat harvesting was predominantly performed from the lower extremity in 43 cases, which showed the largest impact on satisfaction in several lip-related items. Fat harvested from the face was used in 32 cases, followed by fat harvested from other sites in 23 cases. The analysis revealed that the harvesting location had a statistically significant impact on patient satisfaction for several lip-related items. For how well the lips suit the face, the lower extremity harvesting site had the largest effect, with a mean satisfaction change of 1.54 (*p* < 0.001), while the face showed a significant increase (1.20, *p* < 0.001). The fullness of the lower lip showed improvements across different harvesting sites, with the other category having the largest impact (mean change = 1.33, *p* < 0.001), followed by the lower extremity (1.15, *p* < 0.001). For Cupid’s bow, patients who had fat harvested from the other category experienced the greatest increase in satisfaction (mean change = 2.00, *p* < 0.001), followed by the face (1.40, *p* < 0.001).

### Influence of Gender on Satisfaction

Gender had a significant influence on several satisfaction items. For how well the lips suit the face, males showed a greater improvement in satisfaction compared to females (mean change = 1.25 for males vs. 0.93 for females, *p* < 0.001). Similarly, males reported a higher increase in satisfaction for how nice the lips look when smiling (mean change = 1.25, *p* < 0.001) compared to females (mean change = 0.67, *p* < 0.001). Additionally, for fullness of the lower lip, males had a larger increase in satisfaction (mean change = 1.33, *p* < 0.001) than females (mean change = 1.00, *p* < 0.001). However, females showed a slightly higher improvement in satisfaction for the style of the lips (mean change = 1.15 for females vs. 1.00 for males, *p* < 0.001).

### Influence of Age on Satisfaction

Age had a statistically significant influence on satisfaction for specific items. For how well the lips suit the face, a positive correlation was found between age and satisfaction change (*r* = 0.18, *p* = 0.037), indicating that older patients experienced greater improvements in satisfaction. A similar trend was observed for the size of the lips, where a moderate positive correlation (*r* = 0.23, *p* = 0.018) suggested that older patients were more satisfied with the change in lip size postoperatively. Conversely, for the fullness of the upper lip, a moderate negative correlation (*r* = − 0.28, *p* = 0.004) indicated that younger patients were more likely to report greater satisfaction with this aspect.

### Influence of Injected Volume on Satisfaction

The total volume of fat injected had a significant impact on satisfaction for several items. For how nice the lips look when smiling, a positive correlation (*r* = 0.13, *p* = 0.045) was found, suggesting that higher injection volumes led to greater satisfaction with this item. Similarly, for the fullness of the upper lip, a positive correlation (*r* = 0.14, *p* = 0.038) indicated that patients who received larger injection volumes reported higher satisfaction. No significant correlations were found between injected volume and satisfaction changes for other items.

### Influence of Preparation Method on Satisfaction

**Fig. 1:**
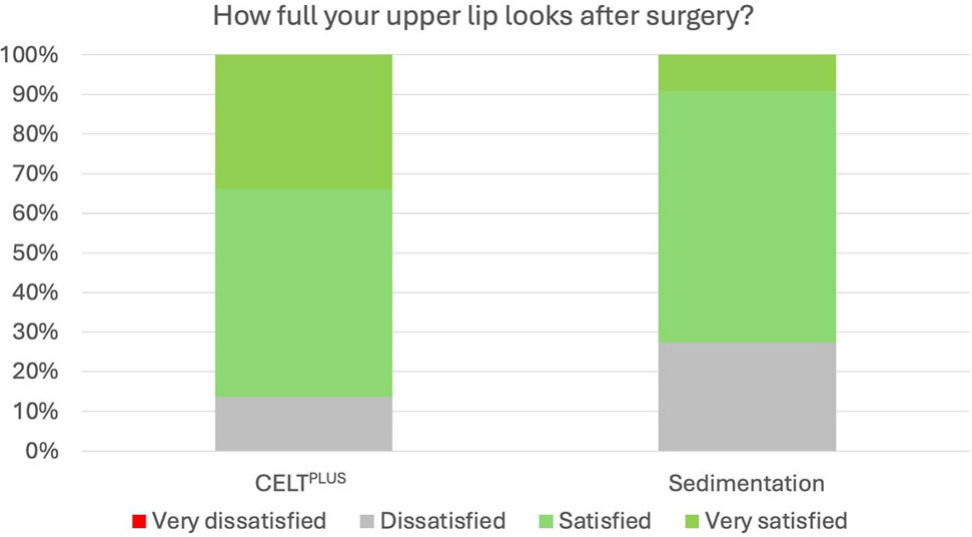
Percentage of very satisfied, satisfied, unsatisfied and very unsatisfied patients in regards to their upper lip fullness after injection with CELT^PLUS^ technique and classical sedimentation

**Fig. 2:**
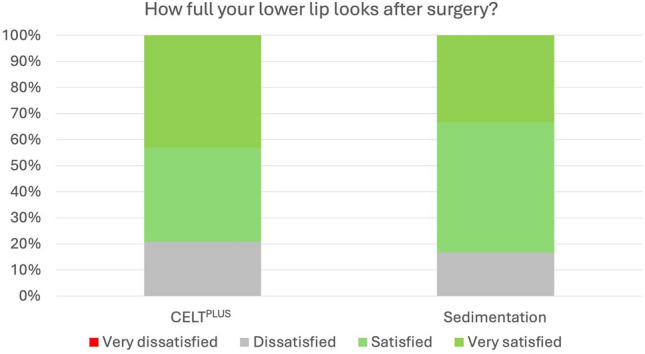
Percentage of very satisfied, satisfied, unsatisfied and very unsatisfied patients in regards to their lower lip fullness after injection with CELT^PLUS^ technique and classical sedimentation

**Fig. 3:**
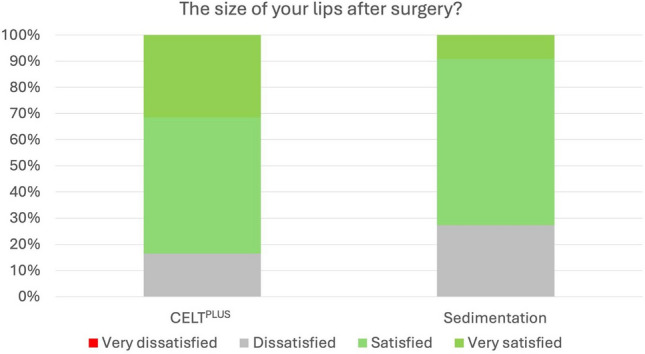
Percentage of very satisfied, satisfied, unsatisfied and very unsatisfied patients in regards to their lip size after injection with CELT^PLUS^ technique and classical sedimentation

When comparing the CELT^PLUS^ preparation method to the combined sedimentation/filtration group, significant differences in patient satisfaction were found across all items. For the shape of the lower lip, the CELT^PLUS^ method resulted in a greater increase in satisfaction compared to the combined methods, with a *p* value of *p* < 0.001. Similarly, for how well the lips suited the face, patients who underwent the CELT^PLUS^ method reported significantly higher satisfaction (*p* < 0.001). For how nice the lips looked when smiling, the CELT^PLUS^ method again produced greater satisfaction, showing a significant difference compared to sedimentation/filtration (*p* < 0.001). Additionally, satisfaction with the fullness of the lower lip was higher in the CELT^PLUS^ group, with a *p* value of *p* < 0.001. The style of the lips, shape of the upper lip, and Cupid’s bow all saw significantly greater satisfaction among patients who had undergone the CELT^PLUS^ preparation method, with all *p* values less than 0.001. The size of the lips, outer corners of the lips, and fullness of the upper lip also showed similar trends, with the CELT^PLUS^ method resulting in higher satisfaction across these features (all *p* < 0.001) (Figs. 1, 2, 3).

## Discussion

This study aimed to retrospectively assess patient satisfaction after lip augmentation using autologous fat grafting. The investigation focused on comparing the CELT^PLUS^ technique with traditional fat preparation methods like sedimentation and filtration. Satisfaction was evaluated using the FACE-Q^TM^ questionnaire, a validated tool widely used in cosmetic surgery to assess patient-reported outcomes. The key variables analyzed included the method of fat preparation, the amount of fat injected, harvesting location, patient demographics (age, gender, smoking status), and any complications. The study provides valuable insights into the long-term satisfaction of patients who underwent lip augmentation with autologous fat grafting.

One of the most important findings was that the CELT^PLUS^ preparation method led to consistently higher levels of patient satisfaction compared to sedimentation and filtration. For all measured aspects, such as the shape of the lips, fullness of the lower lip, and Cupid’s bow, the CELT^PLUS^ method showed a significantly higher increase in satisfaction, with *p* values < 0.001 across all items. This suggests that the CELT^PLUS^ technique, which enriches the harvested fat with adipose tissue-derived stem cells (ADSCs), may promote better fat survival and integration, leading to more consistent and aesthetically pleasing results over time.

**Fig. 4:**
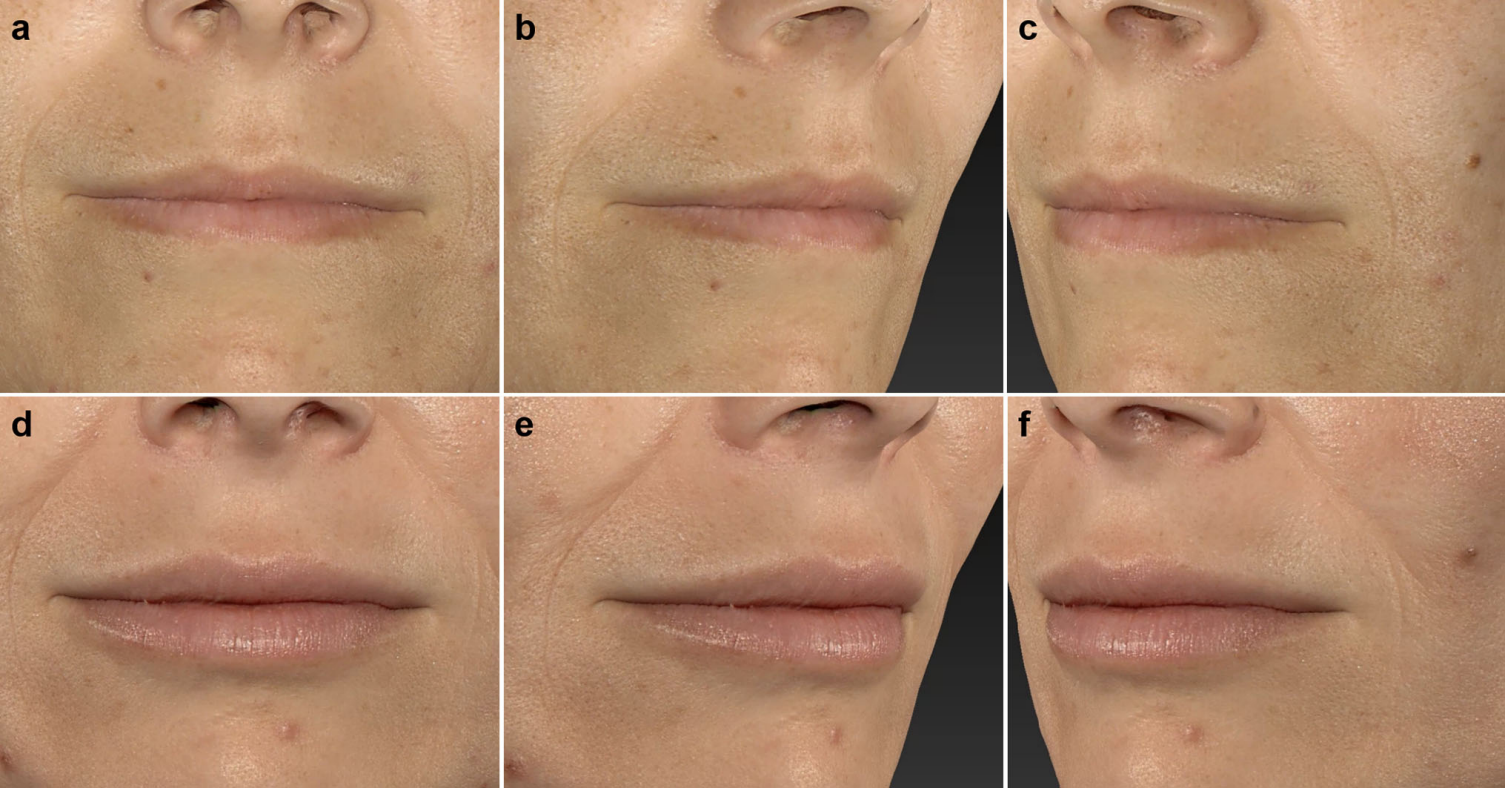
Patient treated with CELT^PLUS^ technique before (**a**–**c**) and after 12 months (**d**–**f**)

**Fig. 5:**
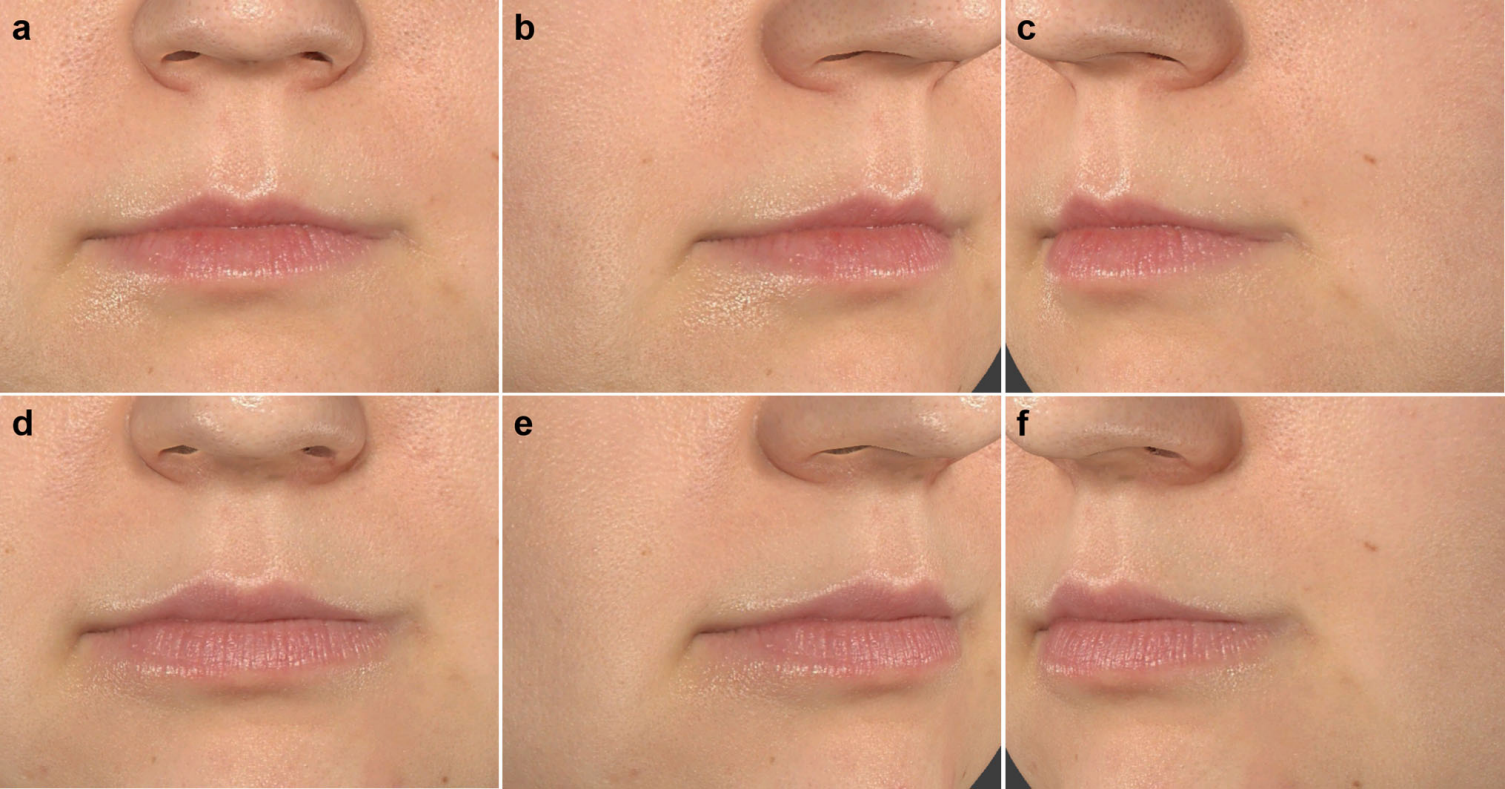
Patient treated with CELT^PLUS^ technique before (**a**–**c**) and after 12 months (**d**–**f**)

**Fig. 6:**
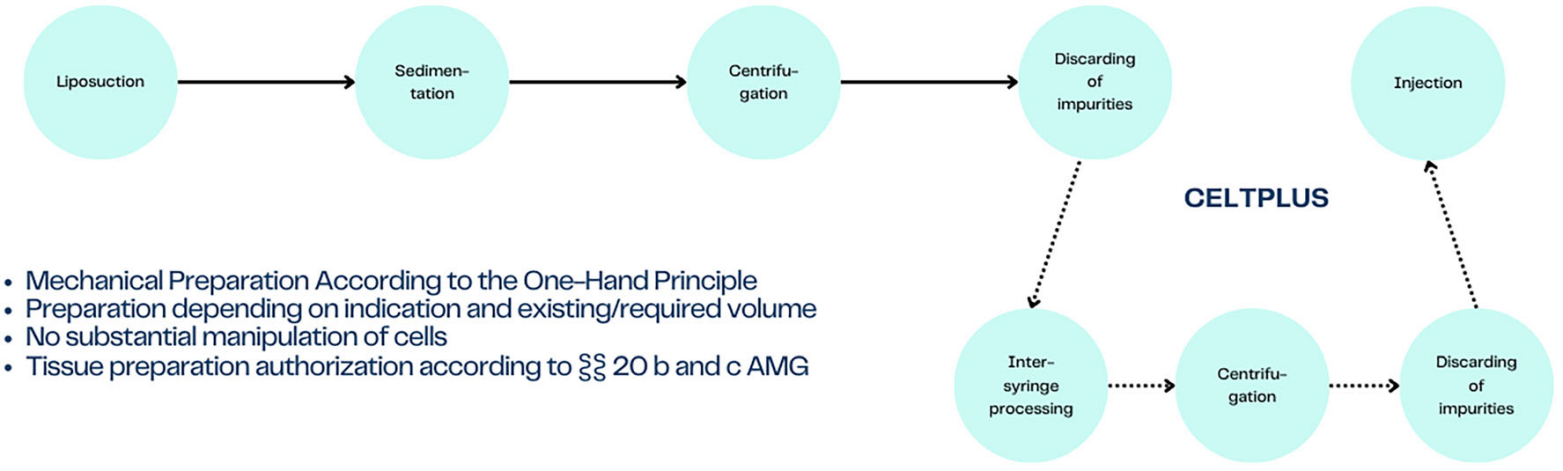
Workflow of the CELT^PLUS^ technique

In terms of gender differences, males generally reported higher satisfaction increases, particularly with how well the lips suited their face and how their lips looked when smiling (*p* < 0.001). Females, on the other hand, showed slightly higher satisfaction with the overall style of the lips, suggesting potential gender-based differences in aesthetic expectations and outcomes. Age also influenced satisfaction, with older patients reporting greater improvements in how their lips suited their face (*p* = 0.037), while younger patients were more satisfied with the fullness of the upper lip (*p* = 0.004). The amount of fat injected positively correlated with satisfaction in several key areas, such as lip fullness and lip appearance when smiling, indicating that a higher volume of injected fat led to better aesthetic outcomes (Figs. 4, 5, 6).

The CELT^PLUS^ technique represents a more refined approach to autologous fat grafting. The process begins with the harvest of fat tissue from the patient, typically from areas such as the abdomen or thighs, using liposuction. Once the fat is extracted, it undergoes processing to separate and concentrate the adipose tissue-derived stem cells (ADSCs). The harvested fat is first purified by sedimentation to remove unwanted components like blood and fluids. It then undergoes mechanical processing like centrifugation and inter-syringe processing to reduce the fat into smaller particles, further enhancing the concentration of viable adipocytes and stem cells. This enriched fat is then re-injected into the lips using small cannulas, ensuring precision and even distribution. The CELT^PLUS^ method is particularly advantageous because the enriched fat improves the survival rate of the grafted tissue, leading to more predictable and stable results over time [10, 12]. This method has demonstrated greater long-term retention of the fat cells, making it a more effective solution for lip augmentation compared to traditional fat grafting techniques.

In lip augmentations, HA fillers are the most commonly used non-permanent solution. Studies on HA fillers report high patient satisfaction, particularly within the first few months following the procedure. For example, satisfaction rates for HA fillers were reported at 85.8/100 at Week 6 on the FACE-Q scale, with 99.1% of patients pleased with the results. However, by Month 18, satisfaction rates dropped to 69.1%, as HA fillers require regular touch-ups to maintain the desired effect [13]. In contrast, CELT^PLUS^ fat grafting offers a more permanent solution with superior long-term retention, reducing the need for frequent procedures. The enriched fat used in the CELT^PLUS^ technique shows greater stability in dynamic areas like the lips, likely contributing to the higher patient satisfaction levels observed in this study. Patients treated with CELT^PLUS^ also benefit from the natural look and feel of autologous fat, as opposed to synthetic fillers, which can sometimes create a less natural appearance over time as they degrade.

Despite the promising results, this study has several limitations. First, the lack of three-dimensional (3D) imaging to objectively assess changes in lip volume and shape is a significant drawback. Objective volumetric assessments could provide a more precise measurement of the outcomes, allowing for a clearer comparison between pre- and postoperative results. The current study does not include longitudinal assessments of fat graft retention or resorption rates. However, future IRB-approved research will utilize three-dimensional surface imaging to objectively evaluate these parameters. This forthcoming work will provide a deeper understanding of volume retention over time. Additionally, the study relied solely on subjective patient-reported outcomes via the FACE-Q^TM^ questionnaire, which, while validated, does not incorporate an objective scale for measuring changes in lip volume. The use of validated volumetric scales would have strengthened the findings and allowed for a more comprehensive analysis of fat retention and lip contour changes over time. A key limitation of this study is the retrospective nature of data collection, which involved assessing patient satisfaction through a single post-procedure questionnaire without predefined time intervals. While this approach provides valuable insights, it limits the ability to analyze satisfaction trends over time. Future prospective studies should include multiple data collection points to address this gap. Despite these limitations, the study has several strengths. The use of the CELT^PLUS^ technique provides valuable insights into the benefits of stem cell-enriched fat grafting compared to traditional methods like sedimentation and filtration. Additionally, the relatively large sample size enhances the robustness of the findings, allowing for meaningful statistical comparisons across different preparation methods. Another strength is the comparison of multiple preparation techniques, which is rarely done in similar studies. This comparison highlights the clinical advantages of the CELT^PLUS^ method, which shows higher satisfaction levels and better fat retention, making it a potentially superior technique for patients seeking long-term lip augmentation results.

## Conclusion

This study demonstrates that CELT^PLUS^ fat grafting offers superior patient satisfaction compared to traditional fat grafting techniques. Its long-term results are also comparable to, if not better than, HA fillers, which require more frequent touch-ups. Future studies incorporating 3D volumetric assessments and validated lip volume scales would further substantiate these findings and provide a more comprehensive evaluation of the effectiveness of the CELT^PLUS^ technique in lip augmentation.

## References

[1] Triana L, Palacios RM, Huatuco GC, Liscano E. Correction: trends in surgical and nonsurgical aesthetic procedures: a 14-year analysis of the International Society of Aesthetic Plastic Surgery—ISAPS. Aesthet Plast Surg. 2024;48(21):4601–4601. 10.1007/s00266-024-04355-w. 10.1007/s00266-024-04355-w 39225818

[2] Tabassum N, Jasthi VC, Al Salem A, et al. Perspectives and challenges in lip rejuvenation: a systematic review. Eur Rev Med Pharmacol Sci. 2023; 27(19). 10.26355/eurrev_202310_33929 37843317

[3] Tonnard PL, Verpaele AM, Ramaut LE, Blondeel PN. Aging of the upper lip: part II. Evidence-based rejuvenation of the upper lip—a review of 500 consecutive cases. Plast Reconstr Surg. 2019;143(5):1333–42. 30789473 10.1097/PRS.0000000000005589

[4] Jalalabadi F, Lisiecki JL, Chiodo MV, Rohrich RJ. Lip lifting: the missing link in central facial rejuvenation. Plast Reconstr Surg. 2024;154(1):79e–84e. 37220218 10.1097/PRS.0000000000010735

[5] Ehlinger-David A, Gorj M, Braccini F, et al. A prospective multicenter clinical trial evaluating the efficacy and safety of a hyaluronic acid-based filler with Tri-Hyal technology in the treatment of lips and the perioral area. J Cosmet Dermatol. 2023;22(2):464–72. 35718985 10.1111/jocd.15169 PMC10087550

[6] Czumbel LM, Farkasdi S, Gede N, et al. Hyaluronic acid is an effective dermal filler for lip augmentation: a meta-analysis. Front Surg. 2021;8:681028. 34422892 10.3389/fsurg.2021.681028 PMC8377277

[7] Strong AL, Rohrich RJ, Tonnard PL, Vargo JD, Cederna PS. Technical precision with autologous fat grafting for facial rejuvenation: a review of the evolving science. Plast Reconstr Surg. 2024;153(2):360–77. 37159906 10.1097/PRS.0000000000010643

[8] Ince B, Zuhour M, Kadiyoran C, Avunduk MC, Dadaci M. A comparison between hyaluronic acid filler and dermofat grafts with or without tie-over dressing for lip augmentation. Dermatol Surg. 2024;50(1):52–8. 37994437 10.1097/DSS.0000000000003995

[9] Wu M, Li Y, Wang Z, et al. Botulinum toxin A improves supramuscular fat graft retention by enhancing angiogenesis and adipogenesis. Dermatol Surg. 2020;46(5):646–52. 31415259 10.1097/DSS.0000000000002106

[10] Prantl L, Eigenberger A, Reinhard R, Siegmund A, Heumann K, Felthaus O. Cell-enriched lipotransfer (CELT) improves tissue regeneration and rejuvenation without substantial manipulation of the adipose tissue graft. Cells. 2022;11(19):3159. 36231121 10.3390/cells11193159 PMC9563290

[11] Gontijo-de-Amorim NF. Fat grafting for facial contouring using mechanically fraction-enriched stromal lipotransfer vascular. Clin Plast Surg. 2019;47:99. 31739903 10.1016/j.cps.2019.08.012

[12] Eigenberger A, Felthaus O, Schratzenstaller T, Haerteis S, Utpatel K, Prantl L. The effects of shear force-based processing of lipoaspirates on white adipose tissue and the differentiation potential of adipose derived stem cells. Cells. 2022;11(16):2543. 36010620 10.3390/cells11162543 PMC9406387

[13] Müller DS, Grablowitz D, Krames-Juerss A, Worseg A. Lip augmentation with Saypha LIPS Lidocaine: a postmarket, prospective, open-label, randomized clinical study to evaluate its efficacy and short- and long-term safety. Aesthet Surg J. 2024;45(1):84–97. 10.1093/asj/sjae149. 39167667 10.1093/asj/sjae149 PMC11634382

